# Giant clam (*Tridacna*) distribution in the Gulf of Oman in relation to past and future climate

**DOI:** 10.1038/s41598-022-20843-y

**Published:** 2022-10-03

**Authors:** Markus Reuter, Philipp M. Spreter, Thomas C. Brachert, Regina Mertz-Kraus, Claudia Wrozyna

**Affiliations:** 1 Institute of Geography and Geology, University of Greifswald, Friedrich-Ludwig-Jahn-Straße 17a, 17489 Greifswald, Germany; 2 Institute of Geophysics and Geology, Leipzig University, Talstraße 35, 04103 Leipzig, Germany; 3 Institute of Geosciences, Johannes Gutenberg University Mainz, Johann-Joachim-Becher-Weg 21, 55128 Mainz, Germany

**Keywords:** Biogeography, Climate-change impacts, Stable isotope analysis

## Abstract

The Oman upwelling zone (OUZ) creates an unfavorable environment and a major biogeographic barrier for many coral reef species, such as giant clams, thus promoting and maintaining faunal differences among reefs on the east and west side of the Arabian Peninsula. We record the former existence of *Tridacna* in the Gulf of Oman and review its stratigraphic distribution in the Persian Gulf to provide new insights on the connectivity of coral reef habitats around southern Arabia under changing climate and ocean conditions. Fossil shells were carbon-14 dated and employed as sclerochronological proxy archives. This reveals that the Omani population represents a last glacial colonization event during the Marine Isotope Stage 3 interstadial under colder-than-present temperatures and variable upwelling intensity linked to Dansgaard-Oeschger climate oscillations. It was favored by temperatures just above the lower threshold for the habitat-forming reef coral communities and instability of the upwelling barrier. We conclude that the distribution of *Tridacna* in the northern Arabian Sea is generally limited by either strong upwelling or cool sea surface temperature under gradually changing climate conditions at the interglacial-glacial scale. Opportunities for dispersal and temporary colonization existed only when there was a simultaneous attenuation of both limiting factors due to high-frequency climate variability. The OUZ will unlikely become a future climate change refuge for giant clams because they will be exposed either to thermal stress by rapid anthropogenic Indian Ocean warming or to unfavorable upwelling conditions.

## Introduction

Giant clams (Cardiidae: Tridacninae) are iconic animals of Indo-Pacific coral reefs, which help to maintain the overall reef biodiversity and functionality by contributing to the reef carbonate structure and serving various ecosystem functions^1–4^. Because of their great importance for community dynamics, stability and diversity, conservation and preservation of such “key(stone)” species have shifted into the focus of recent conservation strategies^2^.

Unlike most other bivalves, but similar to most reef-building corals, the species of the Tridacninae host symbiotic dinoflagellate photoautotrophs of the Symbiodinaceae family^3,4^. These symbionts contribute significantly to the energy budget of the host and encourage the large shell growth^3,4^, with the largest shells reaching > 1 m length^5^. Light availability is thus essential for giant clam growth and survival^3,4^. When ambient seawater gets too warm for too long, giant clams release their symbionts thereby turning white and starving, a process known as bleaching^4,5^. Additionally, elevated temperature reduces fertilization success in giant clams^5,6^. High *p*CO_2_ seawater conditions can also alter the symbiont density and photosynthetic yield that affect the biomineralization process^5^. Giant clams are therefore expected to particularly suffer from human-caused ocean warming and acidification^4–6^.

**Figure 1:**
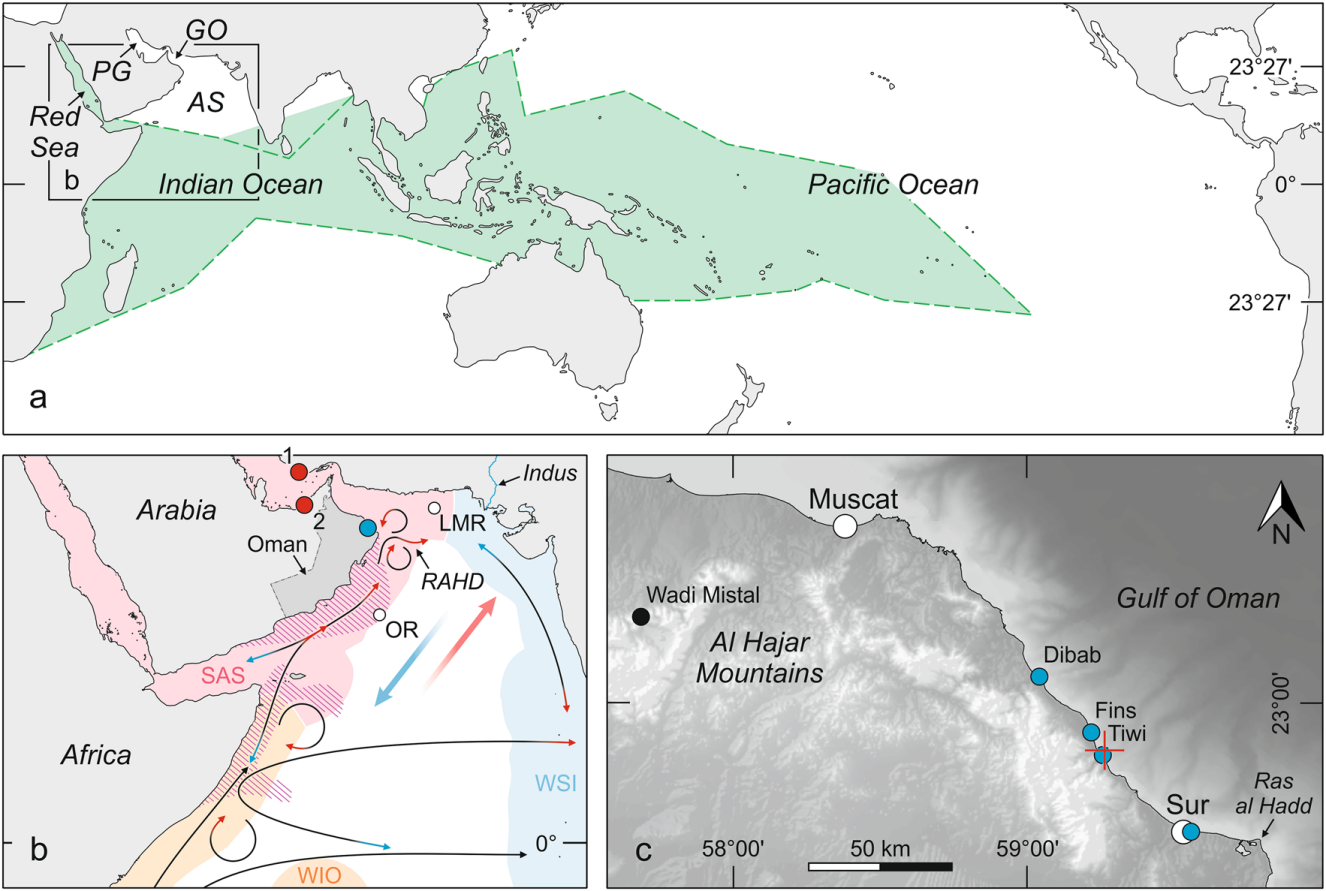
Giant clam biogeography and study area. (**a**) Present-day geographical distribution of the Tridacninae (green area) and *Tridacna maxima* (dashed green line) in the Indo-West Pacific region^5^ (*AS* Arabian Sea, *GO* Gulf of Oman, *PG* Persian Gulf). (**b**) Detailed map indicating last interglacial (red dots) and last glacial (blue dot) *Tridacna* occurrences in the Persian Gulf (1: Kish Island^22^, 2: Abu Dhabi^7^) and Gulf of Oman, and recent coral biogeographic provinces of the western Indian Ocean (*WIO* Western Indian Ocean Province, *SAS* Somali-Arabian Seas Province, *WSI* West and South India Province)^8^. Seasonal wind directions are shown by red (SWM, summer) and blue (NEM, winter) large arrows. The surface circulation in the Arabian Sea is indicated by thin arrows (*RAHD* Ras al Hadd oceanic dipole). Seasonally reversing currents are marked by red (SWM) and blue (NEM) arrowheads. Coastal upwelling zones during the SWM season are highlighted by purple hatching. White dots indicate the locations of SWM multi-proxy stacks (*OR* Owen Ridge^48^, *LMR* Little Murray Ridge^49^). (**c**) Bathymetric and topographic map of northeastern Oman (GEBCO 2020 bathymetric data, https://www.gebco.net) showing the locations of fossil *Tridacna* sites considered in this study (blue dots), the measurement location (red cross) of recent SST data (Supplementary Fig. S3), and the location of the fluvio-lacustrine record of MIS3 precipitation pattern from Wadi Mistal^29^ (black dot). The maps were created using the CoralDraw Graphics Suite 2017 (https://www.coreldraw.com) and QGIS 2.18.15 (https://www.qgis.org) software.

The present geographical distribution of the Tridacninae is throughout the tropical Indo-Pacific region and extends northward to the northern Red Sea and subtropical Japan^5^ (Fig. 1a). But within this latitudinal range, they are remarkably absent from the northern Arabian Sea, Gulf of Oman and Persian Gulf^5,7^ (Fig. 1a), despite the presence of potential shallow-water habitats associated with reef coral communities. In terms of recent coral biogeography, the Red Sea, Gulf of Aden, Gulf of Oman, and Persian Gulf are defined as distinct ecoregions that are grouped together in a single province, which is clearly distinguished from the adjacent Western Indian Ocean Province and West and South India Province^8^ (Fig. 1b). The eastern boundary of this so-called Somali-Arabian Seas Province^8^ corresponds to the freshwater barrier formed by the discharge of the Indus River^9^ and its southern boundary corresponds to the Somali upwelling system^10,11^ (Fig. 1b). Furthermore, faunal connectivity within the Somali-Arabian Seas Province is impaired for many coral reef-dependent species by the seasonally reversing Indian monsoon system that drives upper-ocean circulation and induces one of the world’s largest upwelling systems off south-east Arabia^12–14^ (Fig. 1b). During northern hemisphere winter the northeast monsoon (NEM) blows away from the Asian continent and thus the ocean circulation in the Arabian Sea is counter-clockwise (Fig. 1b), restricting eastward dispersal of larvae from the Red Sea and Gulf of Aden to the Gulf of Oman and Persian Gulf^14^. Larvae released in the Red Sea and Gulf of Aden have a better chance to reach the Omani coast during northern hemisphere summer when the strong winds of the southwest monsoon (SWM) cause the ocean currents to flow clockwise^14^ (Fig. 1b). However, dispersal around the Arabian Peninsula is limited at this time by monsoon-induced coastal upwelling of deep, cold water off southern Oman, which is rich in dissolved inorganic nutrients and carbon dioxide but low in oxygen and pH. This results in a “pseudo-high latitude effect”, which impedes coral growth and reef framework development along the southern Oman coast from Dhofar to Cape Ras al Hadd^12^. In addition, much of the southern Oman coast is sandy and thus unsuitable for coral settlement^12^. This poor habitat continuity is assumed to restrict stepping-stone connectivity for many coral reef-associated biota^13,14^. Offshore Ekman drift further tends to transport planktic larvae within the surface layer into areas unsuitable for settling, thus reducing the larval dispersal and recruitment success^14^.

We describe a fossil range-edge population of *Tridacna maxima* in northeastern Oman. This relative small (< 35 cm) giant clam species dwells in reefs and lagoons, rarely beyond a depth of 10 m owing to its strong reliance on light^5^. It has the most widespread distribution of the Tridacninae today, encompassing almost the entire geographical range of all other species^5^ (Fig. 1a). The diagenetically robust aragonite shell of giant clams is a valuable high-resolution geochemical proxy archive of near-sea surface environmental conditions at the time of growth^15–19^. We report the age of *Tridacna* fossils from four sites in northeastern Oman determined by the ^14^C method and have measured the seasonal variability of geochemical proxies for temperature, ocean primary productivity and photoautotrophy (δ^18^O, δ^13^C, Sr/Ca, Mg/Ca, B/Ca, Ba/Ca) in two dated shells. The aims are to understand: (1) the timing of the ancient range expansion of giant clams into the Gulf of Oman, and (2) the underlying environmental conditions in the range-edge habitat. Such knowledge about range-edge dynamics of “key(stone)” species in the past may help predicting when, where and to what extent species’ distributions can be expected to shift in the face of continuing anthropogenic climate change and thus can contribute towards identifying potential refuges for biodiversity conservation.

## Localities and material

Fossil *Tridacna maxima* shells have been found between 4 and 9 m above sea level (Google Earth Pro altitudes) on raised marine terraces at four localities in northeastern Oman (Dibab, Fins, Tiwi, Sur), which are located within a distance of 75 km (Fig. 1c, Supplementary Table S1). This altitude range assigns the fossil sites to the lower surfaces of Terrace IV of Yuan et al*.*^20^ that have been related to a last glacial sea-level highstand. At each locality, *Tridacna* shells are strongly abraded and preserved disarticulated or fragmented within poorly sorted, coarse bioclastic (corals, mollusks, balanids), pebbly calcarenite or calcarenitic conglomerate (Supplementary Fig. S1).

Typically, the outer shell layer is heavily infested by bioeroders (boring sponges and bivalves; Supplementary Figs. S1d,e, S2) and small coral colonies can be found encrusted on the external surface. In reflected light, vertical cross sections of the shells by height show a distinct macroscopic growth banding of alternating narrow dark and wide light bands (Supplementary Fig. S2). It reflects seasonal variations in shell density and crystallite size (translucency) resulting from growth rate changes in concert with temperature and light variations^17^. Using oxygen stable isotope variations, it was shown that dark bands usually correspond to winters, light bands to summers and one macroscopic dark/light couplet represents one year of shell growth^15^. In addition, multiple very thin macroscopic dark growth bands (herein referred to as “growth lines”) are to be found within the light growth bands of the fossil *Tridacna* shells from Oman (Supplementary Fig. S2).

Two shells, one from Tiwi locality (T-Tiwi1) and one from Fins locality (T-Fins), were selected for geochemical analyses. Both sites are only 6 km apart (Fig. 1c) and located in the same climatic and oceanographic setting. For the other localities, there were no appropriate shells available to produce continuous geochemical records. Only two small shells from juveniles were found at Dibab locality and the shells from Sur locality were diagenetically altered or preserved as fragments, which do not represent the axis of maximum growth.

## Results and discussion

**Figure 2:**
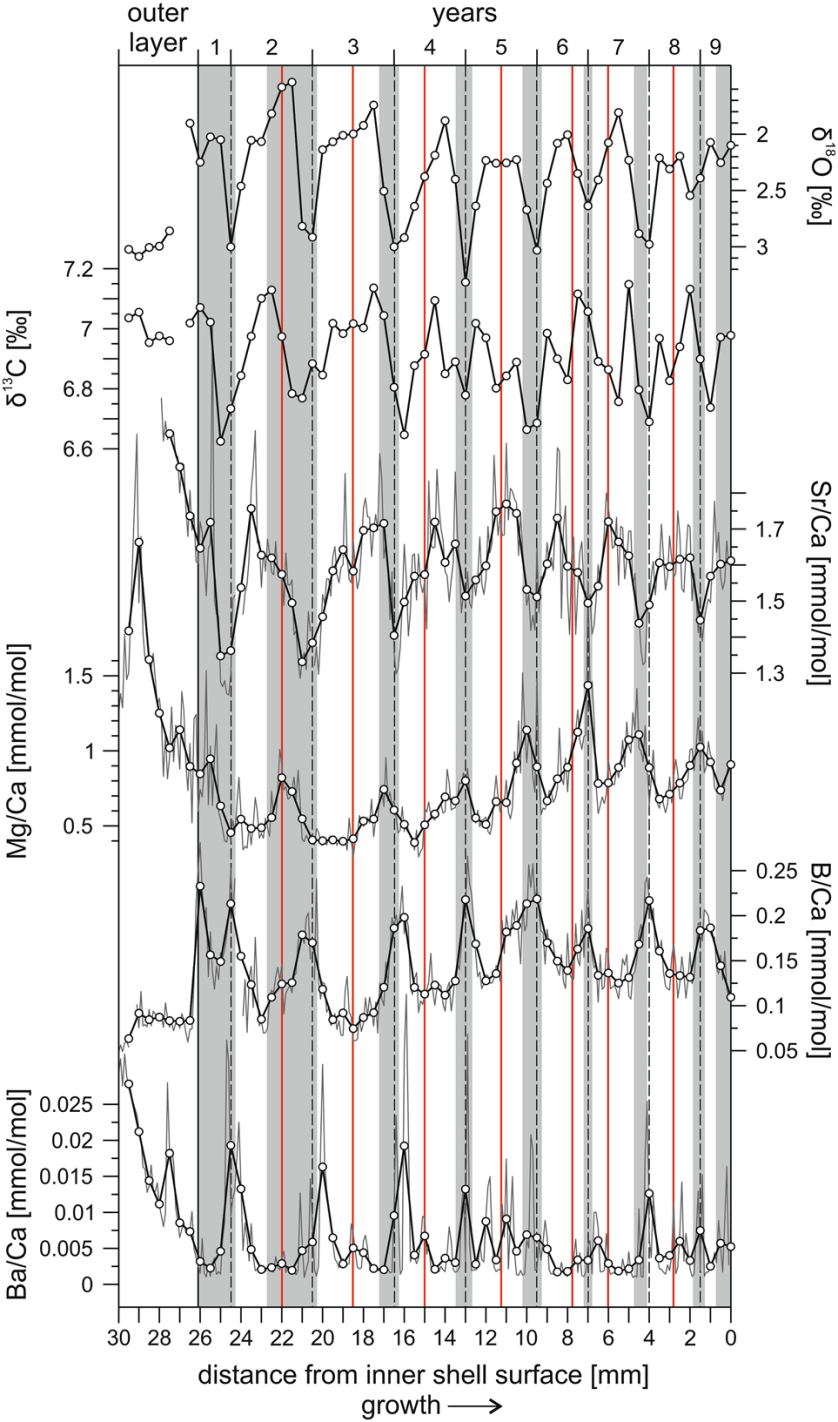
Seasonal variations in the stable isotope and element/Ca composition of T-Tiwi1. Data is plotted as a function of distance from the shell edge. The direction of growth is indicated towards the right (arrow) and most recently secreted layers of carbonate are at 0 mm. Sampled element/Ca data is represented by thin grey lines, with the thicker black line representing the 5 point moving average. Dashed vertical lines define years and vertical red lines indicate upwelling events as deduced from the δ^18^O and δ^13^C data. Vertical grey bars mark the position of dark growth bands as visible along the sampling paths in reflected light (Supplementary Fig. S2a).

**Figure 3:**
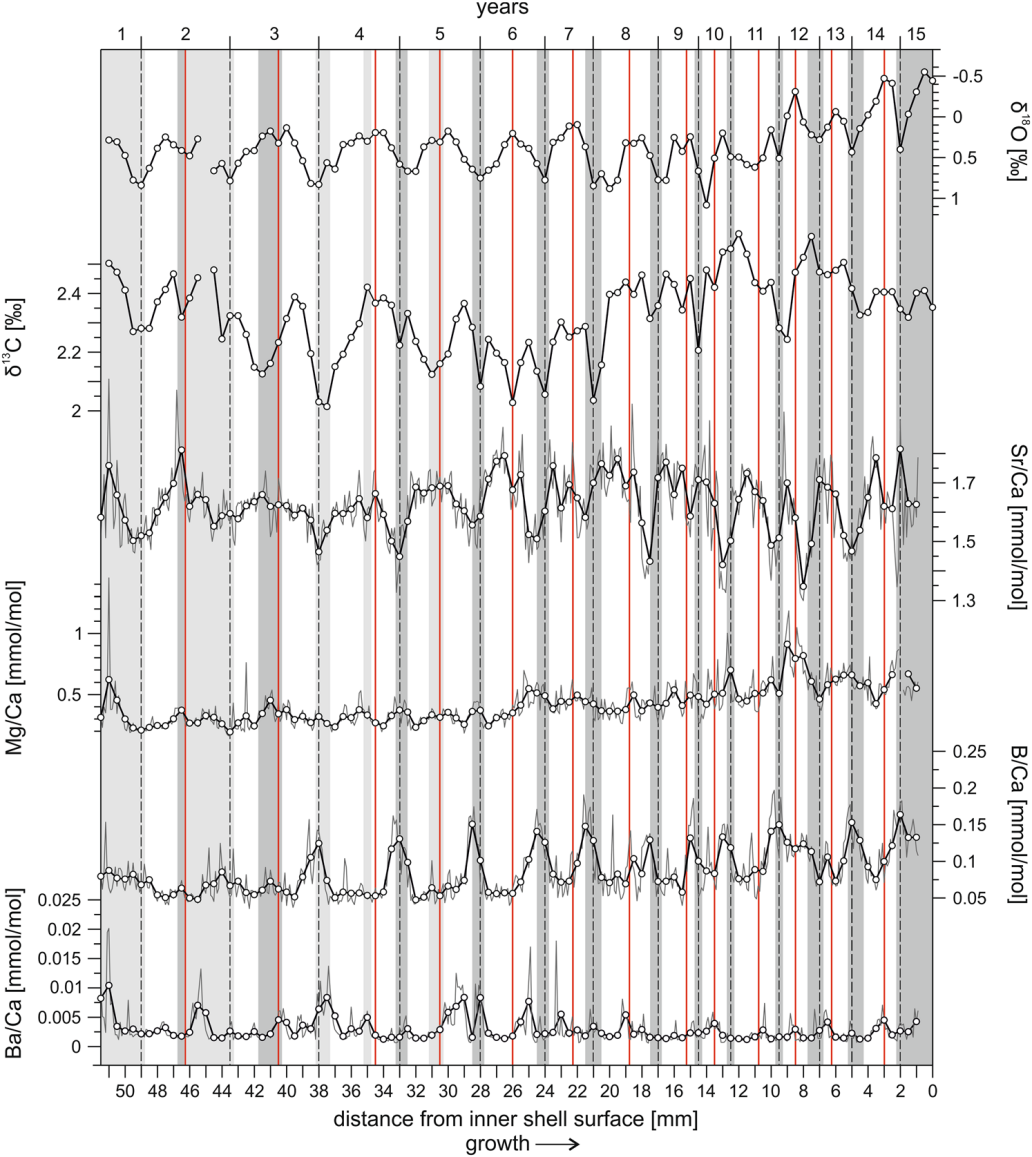
Seasonal variations in the stable isotope and element/Ca composition of T-Fins. Data is plotted as a function of distance from the shell edge. The direction of growth is indicated towards the right (arrow) and most recently secreted layers of carbonate are at 0 mm. Sampled element/Ca data is represented by thin grey lines, with the thicker black line representing the 5 point moving average. Dashed vertical lines define years and vertical red lines indicate upwelling events as deduced from the δ^18^O and δ^13^C data. Vertical grey bars mark the position of dark growth bands as visible along the sampling paths in reflected light (Supplementary Fig. S2b).

Radiocarbon ages determined for nine shell samples from Dibab, Fins, Tiwi and Sur localities range between 34,616 and 47,217 yrs cal BP (Supplementary Table S1). Profiles of oxygen and carbon isotopic ratios, and ratios of Sr, Mg, B and Ba to Ca of the analyzed specimens T-Tiwi1 and T-Fins exhibit regular variations coincident with the primary annual growth banding (Figs. 2, 3). The growth of bivalve shells is not constant and is best described by the von Bertalanffy growth function, where juvenile specimens have an exponential growth while the growth rate in older individuals slows down and stabilizes^21^. This non-linear growth trend is shown by T-Tiwi1 and T-Fins by a decreasing thickness of annual growth increments (Figs. 2, 3). Diagenesis does not follow the von Bertalanffy growth model. Thus, the systematic alignment of geochemical and growth banding patterns provides evidence for an intact geochemical inventory within pristine shell aragonite in the two specimens under study. The results of the geochemical analyses are summarized in Supplementary Table S2, and Supplementary Table S3 represents the statistical relationships among the individual geochemical proxies.

### Occurrences of *Tridacna* in Eastern Arabia

**Figure 4:**
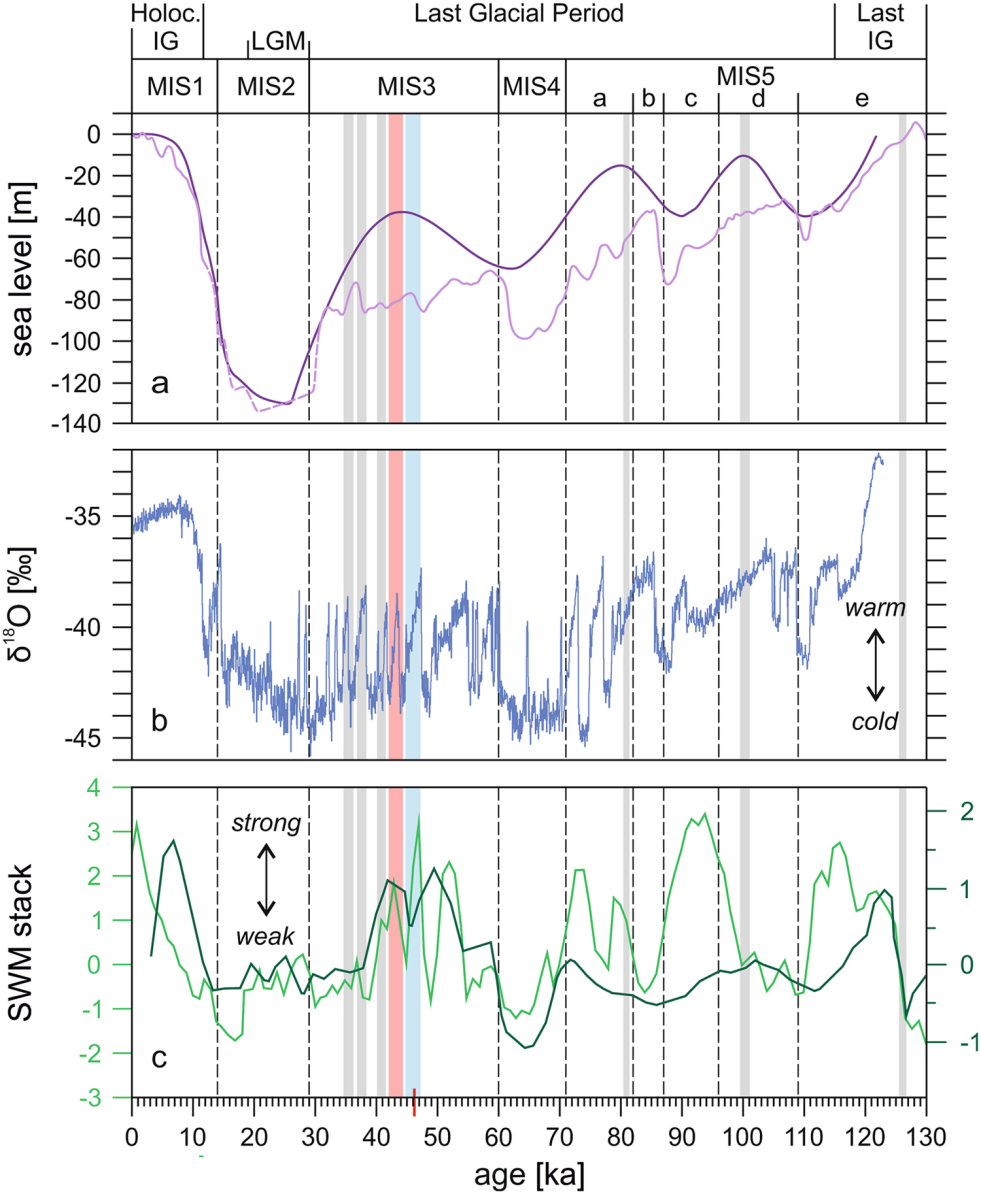
Stratigraphic distribution and climate relationships of late Quaternary *Tridacna* records in the Gulf of Oman and Persian Gulf (vertical bars: blue = T-Tiwi1, red = T-Fins). The red mark on the time axis denotes the maximum age limit of the dating technique applied herein. *IG* interglacial, *LGM* last glacial maximum, *MIS* marine isotope stage. (**a**) Sea-level changes [light purple: global relative sea level (solid line^25^, dashed line^26^), dark purple: global mean sea-level change predicted from ice history simulations^23^]. (**b**) Dansgaard-Oeschger temperature fluctuations as recorded in the North Greenland ice cores^37^. (**c**) SWM multi-proxy stacks from the northern Arabian Sea (dark green: Owen Ridge^48^, light green: Little Murray Ridge^49^; Fig. 1b). The double head arrow indicates SWM strength.

The results from radiocarbon dating (Supplementary Table S1) reveal that the northeastern Oman coast was inhabited by *Tridacna* during the Marine Isotope Stage 3 (MIS3) sea-level highstand ca. 50–35 ka ago (Fig. 4a). So far, *Tridacna* fossils were documented in the region only from the Persian Gulf. Shells of *T. costata*, which is assigned as a synonym of *T. squaminosa*^5^, have been discovered underwater off Abu Dhabi and ^14^C-dated to > 50 ka BP, probably last interglacial (MIS5e)^7^. *Tridacna* from Kish Island in southern Iran were dated by U-Th to 126.1 ± 0.6 ka BP (MIS5e), 100.3 ± 0.8 ka BP (MIS5d) and 80.9 ± 0.5 ka BP (MIS5a)^22^. Thus, the finds in northeastern Oman (Fig. 1c) represent the last occurrence of tridacnids in the gulfs region (Fig. 4), and the only one outside the Persian Gulf (Fig. 1b).

### The role of sea-level changes for *Tridacna* range shifts

The occurrence of *Tridacna* in Oman coincided with an eustatic sea level of 40 m below present sea level (BPSL)^23^ (Fig. 4a), resulting in a massive restriction of the shallow (average depth 35 m) Persian Gulf and hence habitat deterioration and loss for giant clams. For the same sea level, the Gulf of Oman experienced far less drastic changes in environmental conditions because the shelf area remained relatively constant due to its much greater (up to 3,400 m) water depth and steep shelf gradients, and its wide and deep opening to the open ocean ensured an efficient water exchange. The sequence of raised marine terraces along the coast of northeastern Oman yields an extensive Quaternary record of shallow-marine deposition^20,24^. Giant clams are easy to recognize, by virtue of their size, and the thick shells have a high potential for preservation. Nonetheless, their fossils are only found on one marine terrace level. This implies, the Omani population was newly founded during the last glacial period and not the relic of a once-widespread last interglacial population. The Persian Gulf population must have vanished at the latest when the sea-level fall to 60–100-m BPSL during MIS4^23,25^ (Fig. 4a) caused severe habitat loss in the shrinking Gulf. Likewise, the disappearance of tridacnids from the Gulf of Oman was associated with the even greater sea-level fall to 134 m BPSL of the Last Glacial Maximum (29–21 ka BP) during MIS2^26^ (Fig. 4a). The Persian Gulf was not existent at this time and the Red Sea was almost completely cut off from the Arabian Sea^10^. Habitat availability and connectivity related to sea-level changes may therefore account for local extinctions, but it does not explain why giant clams have not sought refuge in the Gulf of Oman during MIS4 and why they did not return during high Holocene sea levels (Fig. 4a). Therefore, special environmental conditions must have prevailed during MIS3, which enabled *Tridacna* to colonize and persist in the Gulf of Oman for a short window of time. Sea surface temperature (SST) is considered as the dominating factor for changing distribution and diversity patterns of tropical coral reefs during the Quaternary climate cycles^27^ and upwelling is the main constraint for shallow-marine biogeographic connectivity around the Arabian Peninsula today^12–14^. Specific information on the seasonal and longer-term variability of SST and upwelling in the range-edge habitat is thus required for understanding the last glacial colonization event of the Gulf of Oman by *Tridacna*.

### Seasonality of the environment

#### Sea surface temperature

As the *Tridacna* shell is secreted in close-to-isotopic equilibrium with ambient seawater, cyclic oxygen isotope variations display changes in temperature and precipitation/evaporation, with shell isotopic composition becoming more positive with decreasing temperature and/or increasing evaporation (salinity)^15–17^. Based on the absence of speleothem growths in the Al Hajar Mountains and a fluvio-lacustrine sediment record from Wadi Mistal (Fig. 1c), a dry semi-arid climate, which was similar to today in terms of the precipitation pattern, is inferred for northeastern Oman during MIS3^28,29^. Furthermore, the rather regular δ^18^O cycles of the fossil shells (Figs. 2, 3) show little intra-annual noise and have no similarity with the “irregular isotope pattern” characterizing intra-annual hydrological effects in sclerochronological datasets from nearshore environments^30^. We therefore suppose that seasonal δ^18^O variations in the shell carbonate were controlled principally by temperature. Consistent with this assumption, the cyclic pattern of δ^18^O in T-Tiwi1 and T-Fins (Figs. 2, 3) reflects the present-day SST seasonality off Tiwi locality (Supplementary Fig. S3, Fig. 1c) with the most positive δ^18^O values, i.e. lowest temperature, during winter. Attenuation of the summer heat peak by upwelled water is indicated as well by the plateau-like and double peak shapes of summer δ^18^O minima in the *Tridacna* records.

#### Photoautotrophy and ocean primary productivity

δ^13^C shows an irregular pattern of ^13^C depletion in winter and associated with upwelling events in summer as derived from δ^18^O (Figs. 2, 3). From the coincidence of significant ^13^C depletions with summer upwelling events and the winter, which is the season of highest cloud cover^31^, least sunshine duration (https://de.climate-data.org/asien/oman/maskat/maskat-2089/) and strongest plankton blooms (high turbidity) in the Gulf of Oman^32^, we deduce that the seasonal δ^13^C drops in the shell carbonate display decreased rates of symbiont photosynthesis and increases in heterotrophy^15^. As the growth rate of *Tridacna* depends on water temperature and photosynthesis of the symbionts^3,33^, periods of cool temperature and low light levels result in decreases of the growth rate and the formation of dark (dense) growth bands^15^. Consistently, dark growth bands in T-Tiwi1 and T-Fins (Supplementary Fig. S2) correspond to winters as inferred from the stable isotope data (Figs. 2, 3). The dark growth lines within the light band of an annual growth increment (Supplementary Fig. S2) are likely related to upwelling filaments of cool and productive water that can be carried into the Gulf of Oman from the Arabian Sea by the Ras al Hadd oceanic dipole eddies (Fig. 1b) accompanied by cloud cover^31,32,34^.

Light-enhanced calcification and elemental transportation processes are supposed to exert major control on diurnal and annual variations of B/Ca, Mg/Ca, Sr/Ca and Ba/Ca ratios within *Tridacna* shells, with high element/Ca ratios relating to low insolation intensities^18,33,35^. The relationships among B/Ca, Mg/Ca, Sr/Ca and Ba/Ca in the herein presented records are inconsistent, however. Just B/Ca und Mg/Ca exhibit a coherent positive correlation in both fossil shells (Supplementary Table S3) with large peaks typically related to the winter growth bands (Figs. 2, 3). This points to a possible role of light for boron and magnesium incorporation, and more specifically reduced light conditions (high cloud cover^31^ and turbidity^32^) in winter. Accordingly, minor peaks in summer (Figs. 2, 3) indicate unfavorable light conditions linked to upwelling filaments. Although, Ba/Ca shows no coherent relationship with B/Ca and Mg/Ca in T-Tiwi1 and T-Fins (Supplementary Table S3), the characteristic peaks are associated with winter growth bands and upwelling events as well (Figs. 2, 3). The concentration of barium in the *Tridacna* shell carbonate, is considered as proxy for ocean primary productivity, being responsible for the Ba/Ca peaks through the ingestion of phytoplankton enriched in Ba^16,19^. Thus, the Ba/Ca peak pattern in the Omani *Tridacna* fossils (Figs. 2, 3) is interpreted to display increases in heterotrophy associated with plankton blooms.

### Long-term variability of sea surface temperature and upwelling

**Figure 5:**
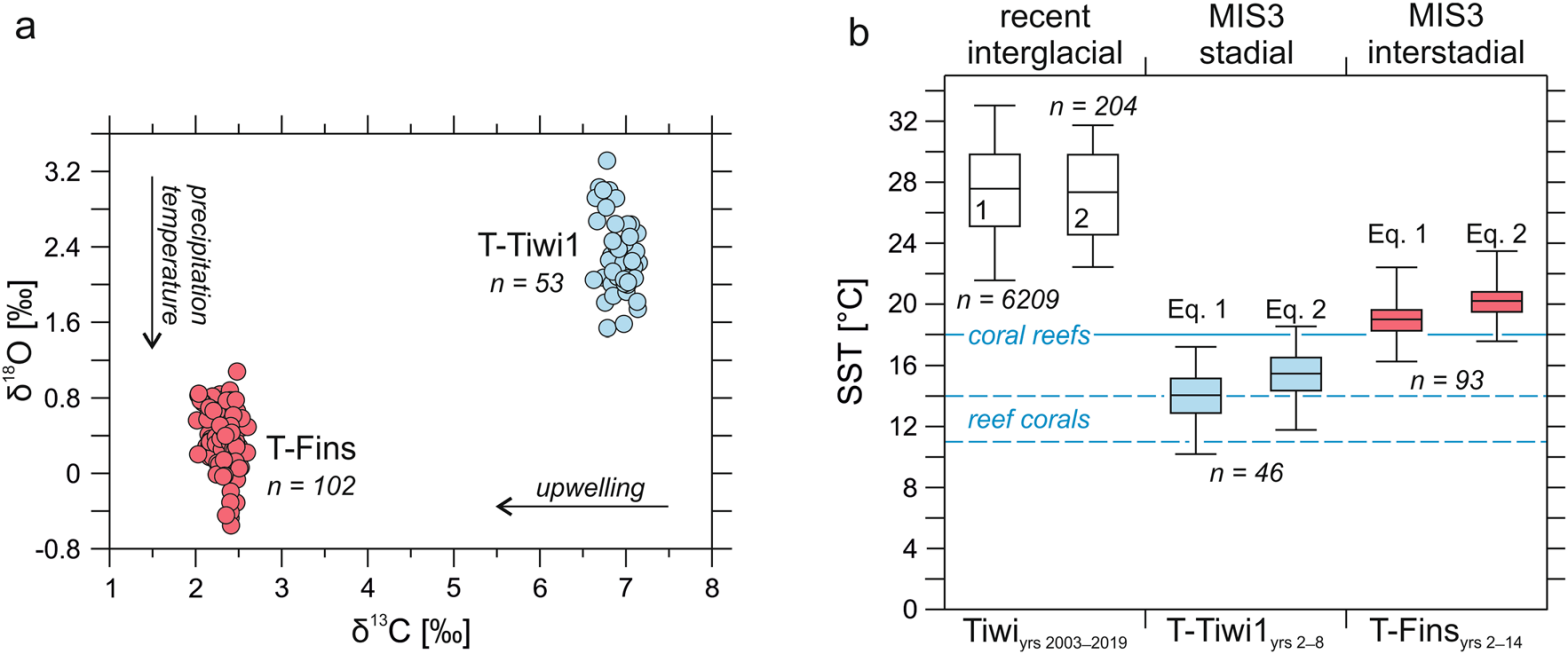
Long-term environmental variability. (**a**) Differences in the δ^18^O and δ^13^C signatures of T-Tiwi1 and T-Fins. Arrows indicate the directions of isotope ratio changes with increasing temperature/precipitation and upwelling strength. (**b**) Variations and trends in annual mean, maximum and minimum sea surface temperature (SST). (1, 2) Recent annual SST variability at Tiwi based on (1) a long-term (2003–2019) satellite-based daily SST dataset (Supplementary Fig. S3) and (2) monthly averages of the daily SST data. MIS3 stadial and interstadial SSTs were calculated from δ^18^O_shell_ values using the equations for *T. maxima*^41^ (Eq. 1) and giant clams^41^ (Eq. 2). The horizontal blue solid line marks the lower temperature threshold for coral reefs^46^ and the horizontal blue dashed lines indicate the lower temperature threshold interval for non-framework forming high-latitude coral communities^47^.

As T-Tiwi1 and T-Fins represent the same sea level^23^ (Fig. 4a), the global ice volume should have left the same signature in both fossil shells. The 1.95‰ heavier mean δ^18^O composition of T-Tiwi1 compared to T-Fins must therefore document conditions of cooler temperature and/or lower precipitation (Fig. 5a, Supplementary Table S2). Upwelling lowers the δ^13^C of surface water dissolved inorganic carbon^36^, which is taken up for photosynthesis by the symbiotic giant clam-dinoflagellate association and by the phytoplankton community that is used as food source for heterotrophic nutrition of the bivalve^3^. The considerably lighter (4.58‰) δ^13^C composition of T-Fins compared to T-Tiwi1 (Fig. 5a, Supplementary Table S2) thus suggests a stronger upwelling intensity regime in the Arabian Sea.

It has been found that the amplitude of Ba/Ca peaks in the *Tridacna* shell displays the amplitude of chlorophyll-α peaks associated with plankton blooms^16,19^. The T-Tiwi1 Ba/Ca profile (Fig. 2) clearly shows that time of peak plankton productivity was the winter such as today in the Gulf of Oman due to convective mixing of the water column^32^. Although summer Ba/Ca peaks are present as well, they are of only low amplitude and occur more randomly. This can be explained with the fact that Oman’s coastal waters are not exposed to SWM winds to the northwest of Ras al Hadd, so the influence of upwelling in the study area is indirect (through the Ras al Hadd oceanic dipole; Fig. 1b) and does not occur regularly^31^. While the height of Ba/Ca summer maximum peaks is equal in T-Tiwi1 (mean 0.009 ± 0.004 mmol/mol) and T-Fins (0.009 ± 0.005 mmol/mol), the Ba/Ca winter maximum peaks are almost fivefold higher in T-Tiwi1 (mean 0.026 ± 0.01 mmol/mol) compared to T-Fins (mean 0.006 ± 0.003 mmol/mol; Figs. 2, 3). From this and in agreement with the stable isotope data we conclude that cooler and eventually also drier winter during lifetime of T-Tiwi1 caused more intense convective mixing, fueling stronger phytoplankton blooms. Accordingly, warmer winter temperatures and enhanced SST cooling in summer by upwelling must be responsible for the subdued amplitude of annual δ^18^O cycles in T-Fins (Supplementary Table S2).

The differences in temperature/precipitation and the intensity of winter convective mixing and summer upwelling among T-Tiwi1 and T-Fins point to a millennial-scale climate variability. As recorded in Greenland ice cores, the climate during MIS3 was characterized by rapid transitions between cold stadials and warm interstadials known as Dansgaard-Oeschger (D-O) events, which occur with a frequency of 2–3 ka and amplitudes close to the glacial-interglacial changes^37^ (Fig. 4b). Although, the key mechanisms triggering these abrupt climate transitions remain elusive, it appears that variations in the strength of the Atlantic meridional overturning circulation (AMOC) were involved^38^. D-O events have been correlated with millennial-scale climate variations worldwide^38^ and also SST, primary productivity and the fluvial versus aeolian sediment discharge in the northern Arabian Sea varied in concordance with D-O oscillations, indicating an apparent linkage between the climate of the Arabian Sea with that of the North Atlantic^39,40^. D-O interstadials were characterized by higher SSTs and ocean primary productivity and show a fluvial signature, which indicates more humid conditions leading to enhanced runoff and transport of suspended load by the Indus River to the ocean^39,40^. D-O stadials, on the other hand, reveal an increased aeolian contribution that at least partly originated from the Arabian Peninsula^39,40^. This has led to the conclusion that the colder D-O stadials were characterized by decreased SWM intensity and by increased northwesterly wind strength and aridity over Arabia^39,40^. Accordingly, T-Tiwi1 might represent a relatively cool and dry D-O stadial, while T-Fins documents warmer and more humid interstadial conditions.

### Sea surface temperature estimations

The use of oxygen isotope ratios is an established method to infer palaeotemperatures from *Tridacna* shell carbonate^16,41^. We apply Eq. (1) defined for *Tridacna maxima*^41^ and Eq. (2) for giant clams^41^ to estimate palaeo-water temperature:

1 $$T= -\,3.96 \pm 0.59\times \Delta {\delta }^{18}\text{O} + 20.12\pm 0.65$$

2 $$T= -\,3.79 \pm 0.65\times \Delta {\delta }^{18}\text{O} + 21.31\pm 0.93$$

where T is the seawater temperature in degree Celsius and Δδ^18^O is the difference between the stable isotopes ratio of the shell and of the seawater (δ^18^O_shell_ $- \delta$ ^18^O_seawater_) in VPDB. The combined measurement of Sr/Ca or Mg/Ca in biogenic calcite and aragonite and stable oxygen isotopes on the same material have been widely employed to calculate the δ^18^O_palaeoseawater_ by subtracting the SST contribution inferred from Sr/Ca or Mg/Ca from δ^18^O^16,31,42,43^. However, there is no significant correlation between Sr/Ca and δ^18^O in both records (Supplementary Table S3), indicating that Sr/Ca ratio in the shell aragonite was not thermodynamically controlled. The T-Tiwi1 Mg/Ca record bears also no relation with δ^18^O, while the correlation is weakly negative in T-Fins (Supplementary Table S3). These poor and inconsistent relationships suggest that Mg/Ca ratios in the fossil shells as well do not record temperature. Therefore, we assume a δ^18^O_seawater_ between 0.3 and 1.0‰ for Δδ^18^O estimation as reconstructed for the eastern Arabian Sea during MIS3^42^. It has been indicated that cool periods during the late Quaternary caused a more positive evaporation-precipitation balance in this region and higher δ^18^O_seawater_ values due to a weaker SWM compared to warm periods^42^. Quantitative calcification temperature reconstructions using a δ^18^O_seawater_ of 1.0‰ (SMOW) may thus be a better approximation for T-Tiwi1, whereas a δ^18^O_seawater_ of 0.3‰ (SMOW) is better suited to approximate absolute water temperatures during lifetime of T-Fins. The δ^18^O_seawater_ reported in SMOW is corrected for conversion to VPDB as follows:

3 $${\delta }^{18}\text{O }\left(\text{VPDB}\right)= {\delta }^{18}\text{O }\left(\text{SMOW}\right)- C$$

where *C* is a constant that is equal to 0.2 following Elliot et al.^16^. The temperatures thus obtained for the last glacial *Tridacna* are cooler than the coastal sea surface temperature at Tiwi today, whereas T-Tiwi1 (D-O stadial) indicates lower temperatures than T-Fins (D-O interstadial; Fig. 5b). This is consistent with alkenone- and Mg/Ca-derived SST reconstructions^44^ and simulations for the last glacial climate^45^ that find evidence for enhanced cooling of the Arabian Sea. In this context, it worth mentioning that fertilization experiments indicate that *T. maxima* can evolve temperature-specific gamete release strategies to reproduce under cooler conditions^6^. Most interestingly, the temperatures for T-Fins are close to the generally accepted 18 °C minimum temperature threshold for coral reef growth^46^, while the temperatures for T-Tiwi1 are around the low temperature threshold known for non-framework forming high-latitude reef coral communities in Japan (14–11 °C)^47^ (Fig. 5b).

### Reasons underlying past *Tridacna* range expansions

SWM stacks from the northern Arabian Sea^48,49^ (Fig. 1b), which integrate proxies for marine productivity (foraminifera assemblages, biogenic opal, barium, bromine) and denitrification (δ^15^N) within the oxygen minimum zone (i.e. upwelling) as well as for terrigenous sediment input (i.e. precipitation), indicate a weaker monsoon during the MIS2 and MIS4 stadials of the last glaciation compared to the last and present interglacials, whereas maximum monsoon strengths during the MIS3 interstadial and the interglacials were comparable (Fig. 4c). Despite this, the occurrence of *Tridacna* in northeastern Oman was apparently limited to MIS3 and giant clams had managed to enter the Persian Gulf during the last interglacial when monsoon-induced upwelling in the northern Arabian Sea was strong (Fig. 4c). Our palaeotemperature reconstructions demonstrate that the last glacial population in the Gulf of Oman had persisted close to the minimum temperature for the habitat-building reef corals (Fig. 5b). Because of the relative warmer global^37^ and regional^50^ temperatures under the intermediate glacial climate of MIS3 compared to the preceding (MIS4) and following (MIS2) stadials (Fig. 4b), this implies that low SSTs caused the deterioration of reef coral ecosystems in the Gulf of Oman during the stadials. Accordingly, the only evidence for last glacial coral reef accretion in the Gulf of Oman is from MIS3^51^. Therefore, we consider that low temperatures and the resultant lack of habitats had prevented the gulfs’ colonization by giant clams during stadials of the last glaciation, despite of weak Oman upwelling and Indus freshwater barriers that are both dependent on the strength of SWM. Although the low-resolution monsoon stacks from the Arabian Sea suggest rather high intensity upwelling conditions for the MIS3 interval^48,49^ (Fig. 4c), fluctuations of upwelling intensity related to high-frequency D-O climate oscillations^39,40^ had offered short-term opportunities for *Tridacna* to enter the Gulf of Oman during this episode of relatively warm glacial climate (Fig. 4b). In agreement with this interpretation, the early last glacial occurrences of *Tridacna* in the Persian Gulf also correspond to warmer D-O phases for which a higher sea level and weaker SWM are recorded (Fig. 4).

The last interglacial was one of the warmest periods during the last 800 ka^52^. From analogy with the present global warming it has been reasoned that high temperatures were responsible for a loss of equatorial reef coral biodiversity, as revealed by database occurrences of fossil and recent symbiotic reef corals^27^. Actually, the current anthropogenic warming is not a good analogue for the last interglacial warmth because the primary forcing is from CO_2_ rather than orbital^53^. Directly related to this, the reconstruction of spatiotemporal variability of regional and global SSTs from marine sediment core records revealed that the last interglacial warming had particularly affected extratropical regions, whereas tropical SSTs (including those in the northern Arabian Sea) remained even slightly cooler than the pre-industrial average^52^. This would mean less heat stress for Indian Ocean coral reefs during the last interglacial warmth than during the actual temperature rise. We therefore conclude that SST was not a limitation for the distribution of giant clams in the Arabian Sea during the last interglacial. Even though D-O climate oscillations only go back to the beginning of last glacial^37^ (Fig. 4b), the last interglacial was also characterized by enhanced climate instability relative to the pre-industrial Holocene. It is considered as feedback to episodic Northern Hemisphere ice-sheet melting and associated meltwater pulses in the North Atlantic, which decreased the strength of AMOC^52,54,55^. The Indian monsoon circulation shows a close relationship to AMOC and weakens in response to AMOC weakening^56^. In consequence, limited time windows for the dispersal of *Tridacna* around the Arabian Peninsula could have opened also during the last interglacial through the weakening of monsoon-dependent upwelling along the Oman Margin linked to major Northern Hemisphere melting events. In support of this hypothesis, the first evidence for *Tridacna* in the Persian Gulf (126.1 ± 0.6 ka BP)^22^ strikingly coincides with a prominent outburst flooding event from the melting Laurentide Ice Sheet at around 126 ka BP^54^. For this time, the Arabian Sea monsoon stacks^48,49^ show a marked decline in the strength of SWM (Fig. 4c). In contrast to the last interglacial and MIS3 interstadial, a relatively stable monsoonal upwelling is indicated for the entire Holocene interglacial by sedimentary δ^15^N, acting as an indicator for Arabian Sea denitrification and hence oxygen minimum zone intensity^44^, thus implying a more constant barrier to shallow water marine species dispersal around southern Arabia.

In summary, it can be ascertained that the southern Oman coast, Gulf of Oman and Persian Gulf generally offer unsuitable environments for the dispersal and settlement of giant clams under the prevailing trends of parallel warming (cooling) climate and strengthening (weakening) upwelling over a interglacial–glacial cycle because of the adverse effects of strong upwelling and low temperature on the performance of the bivalve-dinoflagellate holobiont, habitat availability and biogeographic connectivity. Superimposed high frequency climate variability, however, had temporarily opened up narrow environmental windows that allowed *Tridacna* colonization of the Oman and Persian gulfs by inducing weakening of the upwelling barrier during warm climatic episodes of the last interglacial and last glacial. These range-edge populations had failed, however, to endure in the long term probably because giant clams are long-lived and slow to mature and therefore exhibit limited capacity for adaptation to rapid environmental changes^6^.

### Future predictions

SSTs over the Indian Ocean have shown a rapid warming trend over the last several decades^57^, putting increasing stress on the local coral reef ecosystems^58^. With advancing climate change, the OUZ zone is proposed as refuge area for coral reefs in the warming Indian Ocean because upwelling and heat threat coincide, and this overlap reduces the magnitude, frequency and duration of thermal disturbances^59^. But what is not considered in that respect is that the intensity of the Oman upwelling is expected to change under current global warming scenario, even if there is no consensus about the direction of this change. Results from climate modelling indicate an intensification of upwelling in response to the ongoing temperature rise^60^. This would not only further impair the population connectivity between coral reef habitats in the Gulf of Oman ecoregion and the Gulf of Aden and Red Sea ecoregions but could also amplify ocean acidification and its negative impacts on the mineralization and survival of calcifying marine organisms due to the low aragonite saturation state and pH of the upwelled high *p*CO_2_ water^61^. In contrast, δ^18^O and Sr/Ca records of modern and fossil corals from the OUZ zone provide evidence that the Arabian Sea upwelling intensity was very stable over the last millennium and unprecedently declines during the current warming trend^43^. While a reduction of upwelling improves coral reef connectivity within the Somali-Arabian Seas biogeographic province, it lessens the positive cooling effect on the sea surface during summer heat. The coastal waters of Oman will thus not become a suitable climate-change refuge for giant clams and many other calcifying reef species looking to migrate to cooler areas with continuing ocean warming, regardless whether the upwelling strengthens or weakens in future.

## Methods

### Radiocarbon dating

Shell samples from a total of nine specimens (Supplementary Table S1), which preserved the primary growth banding, were sent for radiocarbon dating by accelerator mass spectrometry (AMS) to the laboratory of Beta Analytic (Miami, USA). In order to prevent contamination, shell pieces were carefully selected in avoiding obvious diagenetic textures, borings of endolithic organisms and cracks. After the mechanical removal of attached sediment and weathering crusts, the samples were cleaned in an ultrasonic bath in purified water for 2 min and dried at 38 °C in a drying oven. Furthermore, pre-treatment acid etching was carried out at Beta Analytic to remove any contaminants that appear on the surface of the shell samples. ^14^C ages were calibrated to calendar ages with BetaCal 3.21, which uses the High Probability Density Range Method and Marine13 calibration, and a reservoir correction (Delta-R) of 256 ± 68 as calculated from the website http://calib.org/marine13/ based on local values of Southon et al*.*^62^. 256 ± 68 is the calculated average of two Delta-R values (297 ± 51 and 200 ± 60) given for the same locality (Muscat, N 23° 50′ 00′′, E 58° 60′ 00′′).

### Geochemical analyses

For stable isotope and trace element analyses, a 1.5 cm thick slab was cut in direction from the umbo to the ventral margin along the maximum growth axis from two radiocarbon-dated shells (T-Tiwi1 and T-Fins; Supplementary Fig. S2). The shell slabs were cleaned in an ultrasonic bath in purified water for 2 min and dried at 38 °C in a drying oven. Geochemical analyses were performed along sampling lines that were chosen perpendicular to the shell growth bands in avoiding obvious diagenetic textures, borings of endolithic sponges and bivalves, and cracks (Supplementary Fig. S2).

#### δ^18^O and δ^13^C

To obtain powder samples for isotopic analysis (δ^18^O, δ^13^C) in regular 0.5 mm intervals (drilling depth 0.5 mm) the shell slabs were mounted on a manually operated three-axis precision stage beneath a Proxxon MF 70 micro-mill equipped with a 0.6 mm drill bit. Stable isotope analyses were performed at the Institute of Geophysics and Geology of the Leipzig University in Germany. Carbonate powders were reacted with 105% phosphoric acid at 70 °C using a Kiel IV online carbonate preparation line connected to a MAT 253 isotope ratio mass spectrometer. All δ^18^O and δ^13^C values for carbonate are reported in per mil (‰) relative to the PDB standard, for water against SMOW. Reproducibility was checked by replicate analysis of laboratory standards (internal LAB-standard: δ^18^O_LAB_ =  − 4.97‰ VPDB, δ^13^C_LAB_ = − 3.00‰ VPDB) and was always better than ± 0.08‰ (1σ) for oxygen isotopes (δ^18^O) and better than ± 0.06‰ (1σ) for carbon isotopes (δ^13^C).

#### Trace elements

During two analytical sessions, element concentrations for Sr, Mg, Ba and B were determined at the Institute of Geosciences, Johannes Gutenberg University Mainz (Germany), using an Agilent 7500ce inductively coupled plasma-mass spectrometer (ICP-MS) coupled to an ESI NWR193 ArF excimer laser ablation (LA) system equipped with a TwoVol2 ablation cell. The ArF LA system was operated at a pulse repetition rate of 10 Hz and an energy density of ca. 3 J cm^−2^. Ablation was carried out under a He atmosphere and the sample gas was mixed with Ar before entering the plasma. Measurement spots with a beam diameter of 80 μm were aligned in spot mode with a midpoint distance of 100 μm along transects that run close and parallel to the stable isotope transects. Backgrounds were measured for 15 s prior to each ablation. Ablation time was 30 s, followed by 20 s of wash out. The isotopes monitored were ^11^B, ^25^Mg, ^43^Ca, ^88^Sr and ^138^Ba. Signals were monitored in time-resolved mode and processed using an in-house Excel spreadsheet^63^. Details of the calculations are given in Mischel et al*.*^64^. NIST SRM 610 and 612 were used as calibration material, applying the reference values reported in the GeoReM database^65^ (http://georem.mpch-165mainz.gwdg.de/, Application Version 27) to calculate the element concentrations of the sample measurements. During each session, basaltic USGS BCR-2G, synthetic carbonate USGS MACS-3 and biogenic carbonate (JCp-1-NP for T-Tiwi1, JCt-1-NP for T-Fins) were analyzed repeatedly as quality control materials to monitor precision and accuracy of the measurements as well as calibration strategy. All reference materials were analyzed at the beginning and at the end of a sequence and after ca. 40 spots on the samples. For all materials ^43^Ca was used as internal standard applying for the USGS BCR-2G and MACS-3 the preferred values reported in the GeoReM database, for JCp-1-NP a Ca content of 38.18 wt%^66^, for JCt-1-NP 38.90 wt%^67^, and 39 wt% for the samples. Resulting element concentrations for the control materials together with reference values are provided in Supplementary Table S4. Element concentrations for the samples are converted into molar ratios of Ca, i.e. B/Ca, Mg/Ca, Sr/Ca and Ba/Ca.

### Correlation of sampling transects and data analysis

Corresponding stable isotope and trace element records were matched by using the growth bands as a guide (Supplementary Fig. S2). For T-Fins, the relatively intense bioerosion and associated diagenetic alteration of the shell made it necessary to establish a composite record from three stable isotope and four trace element overlapping sampling transects (Supplementary Fig. S2b). These were unified by best overlap of the oxygen and carbon stable isotope and Sr/Ca and B/Ca profiles, which exhibit a distinct cyclic pattern (Supplementary Fig. S4). To eliminate the effects of the different sampling resolution of the stable isotope and element/Ca records, a 5 point moving average was calculated for each element/Ca dataset and the averaged element/Ca profiles were resampled to 0.5 mm steps using the AnalySeries software^68^. A simple linear regression was then applied to describe the relationships between the geochemical proxies (Supplementary Table S3). Only values from the inner shell layer were used for data analysis^15,17^.

## Supplementary Information

Supplementary Information 1.

Supplementary Information 2.

## Data Availability

All data generated or analysed during this study are included in this published article (and its Supplementary Information files).
