# Supplementary material for: Giant clam (Tridacna) distribution in the Gulf of Oman in relation to past and future climate
Source: Sci Rep. 2022 Oct 3;12:16506. doi: 10.1038/s41598-022-20843-y (PMC9529976; doi:10.1038/s41598-022-20843-y)
Supplement: Supplementary file 1 — Supplementary Information 1. [file 41598_2022_20843_MOESM1_ESM.docx]

**Supplementary Tables and Figures**

for “Giant clam (*Tridacna*) distribution in the Gulf of Oman in relation to past and future climate” by Reuter *et al.*

**Supplementary Table S1.** Localities and radiocarbon ages of fossil *Tridacna maxima* in northeastern Oman.

| locality | GPS coordinates | altitude ASL [m]* | sample | conventional age [yrs BP] ± SD | Delta-R | calibrated age [yrs cal BP], 95.4 % probability |
| --- | --- | --- | --- | --- | --- | --- |
| Dibab | N 23°02'00.01''  E 59°04'52.17'' | 7 | T-DN1 | 36,840 ± 310 | 256 ± 68 | 41,481 – 40,109 |
|  |  |  | T-DN2 | 34,010 ± 210 |  | 38,377 ̶ 36,825 |
| Fins | N 22°53'51.61''  E 59°13'30.21'' | 9 | T-Fins | 40,550 ± 430 |  | 44,355 – 42,825 |
| Tiwi | N 22°50'47.09''  E 59°14'26.46'' | 8 | T-Tiwi1 | 43,320 ± 630 |  | 47,217 – 44,762 |
|  |  |  | T-Tiwi2 | 36,940 ± 310 |  | 41,556 – 40,207 |
|  |  |  | T-Tiwi3 | >43,500 |  | >46,220 |
|  |  |  | T-Tiwi4 | >43,500 |  | >46,220 |
| Sur | N 22°33'54.89''  E 59°35'31.22'' | 4 | T-Sur1 | 33,820 ± 220 |  | 36,261 – 34,616 |
|  |  |  | T-Sur2 | >43,500 |  | >46,220 |

*from Google Earth Pro

**Supplementary Table S2.** Geochemical characteristics of the T-Tiwi1 and T-Fins multi-proxy records.

| proxy record | | | T-Tiwi1 | T-Fins |
| --- | --- | --- | --- | --- |
| stable isotopes | data points [n] | | 59 | 102 |
|  | δ^18^O [‰] | min | 1.54 | -0.47 |
|  |  | max | 3.32 | 1.08 |
|  |  | mean ± SD | 2.32 ± 0.39 | 0.37 ± 0.31 |
|  | δ^18^O cycles | number | 7 (yrs 2 – 8) | 13 (yrs 2 – 14) |
|  |  | min amplitude [‰] | 0.78 | 0.34 |
|  |  | max amplitude [‰] | 1.46 | 0.90 |
|  |  | mean amplitude [‰] ± SD | 1.07 ± 0.21 | 0.63 ± 0.16 |
|  | δ^13^C [‰] | min | 6.63 | 2.01 |
|  |  | max | 7.15 | 2.60 |
|  |  | mean ± SD | 6.91 ± 0.14 | 2.33 ± 0.13 |
| trace elements | data points [n] | | 294 | 507 |
|  | Sr/Ca [mmo/mol] | min | 1.24 | 1.30 |
|  |  | max | 2.27 | 3.16 |
|  |  | mean ± SD | 1.58 ± 0.14 | 1.63 ± 0.13 |
|  | Mg/Ca [mmol/mol] | min | 0.29 | 0.16 |
|  |  | max | 2.13 | 5.78 |
|  |  | mean ± SD | 0.72 ± 0.27 | 0.43 ± 0.30 |
|  | B/Ca [mmol/mol] | min | 0.06 | 0.04 |
|  |  | max | 0.28 | 0.20 |
|  |  | mean ± SD | 0.15 ± 0.04 | 0.09 ± 0.04 |
|  | Ba/Ca [mmol/mol] | min | 0.001 | 0.0006 |
|  |  | max | 0.04 | 0.02 |
|  |  | mean ± SD | 0.005 ± 0.006 | 0.003 ± 0.003 |

| **Supplementary Table S3.** Correlation coefficients (r, lower diagonal half) and p-values (upper diagonal half) from linear regression analysis of the *Tridacna* proxy data. | | | | | | |
| --- | --- | --- | --- | --- | --- | --- |
| T-Tiwi1 | δ^18^O | δ^13^C | Sr/Ca | Mg/Ca | B/Ca | Ba/Ca |
| δ^18^O |  | 0.005 | 0.704 | 0.503 | 0.000 | 0.281 |
| δ^13^C | -0.38 |  | 0.000 | 0.943 | 0.000 | 0.000 |
| Sr/Ca | -0.52 | 0.48 |  | 0.870 | 0.004 | 0.000 |
| Mg/Ca | 0.09 | -0.01 | 0.02 |  | 0.001 | 0.051 |
| B/Ca | 0.68 | -0.50 | -0.39 | 0.43 |  | 0.002 |
| Ba/Ca | 0.54 | -0.48 | -0.46 | -0.27 | 0.41 |  |
|  | | | | | | |
| T-Fins | δ^18^O | δ^13^C | Sr/Ca | Mg/Ca | B/Ca | Ba/Ca |
| δ^18^O |  | 0.008 | 0.942 | 0.000 | 0.360 | 0.922 |
| δ^13^C | -0.26 |  | 0.458 | 0.005 | 0.371 | 0.124 |
| Sr/Ca | 0.01 | -0.08 |  | 0.676 | 0.104 | 0.964 |
| Mg/Ca | -0.36 | 0.28 | 0.04 |  | 0.000 | 0.402 |
| B/Ca | -0.09 | 0.09 | -0.43 | 0.62 |  | 0.314 |
| Ba/Ca | -0.01 | -0.15 | 0.00 | -0.08 | -0.10 |  |

**Supplementary Table S4.** Mean analytical results of element concentrations for quality control materials together with reference values.

| T-Fins | USGS BCR-2G | | | | | | USGS MACS-3 | | | | | | JCt-1-NP | | | | | | |
| --- | --- | --- | --- | --- | --- | --- | --- | --- | --- | --- | --- | --- | --- | --- | --- | --- | --- | --- | --- |
|  | Reference value [µg/g] | 1σ - reference value [µg/g] | Reference | Mean analytical results [µg/g] | 1σ - mean analytical results [µg/g] | Difference from reference value  [%] | Reference value [µg/g] | 1σ - reference value [µg/g] | Reference | Mean analytical results [µg/g] | 1σ - mean analytical results [µg/g] | Difference from reference value  [%] | Reference value [µg/g] | 1σ - reference value [µg/g] | Reference | Mean analytical results [µg/g] | 1σ - mean analytical results [µg/g] | Difference from reference value  [%] |  |
| B/Ca | 6 | 1 | ** | 6.41 | 0.66 | 6.8 | 8.2 | 1.1 | ** | 14.74 | 1.45 | 79.74 | 22.9 | 0.5 | Jochum et al.^S1^ | 20.54 | 1.9 | -10.3 |  |
| Mg/Ca | 21466*** | 543 | ** | 20338 | 700 | -5.3 | 1720 | 100 | ** | 1705 | 60 | -0.9 | 278 | 9 | Jochum et al.^S1^ | 263 | 34 | -5.4 |  |
| Sr/Ca | 342 | 4 * | ** | 340 | 3 | -0.7 | 6640 | 170 | ** | 6672 | 109 | 0.54 | 1380 | 70 | Jochum et al.^S1^ | 1363 | 33 | -1.2 |  |
| Ba/Ca | 683 | 7 * | ** | 690 | 6 | 1 | 59.6 | 1.4 | ** | 60 | 1.8 | 0.7 | 3.73 | 0.48 | Jochum et al.^S1^ | 3.46 | 1.35 | -7.3 |  |

| T-Tiwi1 | USGS BCR-2G | | | | | | USGS MACS-3 | | | | | | JCp-1-NP | | | | | |
| --- | --- | --- | --- | --- | --- | --- | --- | --- | --- | --- | --- | --- | --- | --- | --- | --- | --- | --- |
|  | Reference value [µg/g] | 1σ - reference value [µg/g] | Reference | Mean analytical results [µg/g] | 1σ - mean analytical results [µg/g] | Difference from reference value  [%] | Reference value [µg/g] | 1σ - reference value [µg/g] | Reference | Mean analytical results [µg/g] | 1σ - mean analytical results [µg/g] | Difference from reference value  [%] | Reference value [µg/g] | 1σ- reference value [µg/g] | Reference | Mean analytical results [µg/g] | 1σ - mean analytical results [µg/g] | Difference from reference value  [%] |
| B/Ca | 6 | 1 | ** | 6.52 | 0.47 | 8.6 | 8.2 | 1.1 | ** | 12.6 | 1.1 | 53.4 | 52.4 | 2.2 | Jochum et al.^S1^ | 53.1 | 6.45 | 1.2 |
| Mg/Ca | 21466*** | 543 | ** | 20573 | 262 | -4.2 | 1720 | 100 | ** | 1799 | 39 | 4.6 | 867 | 23 | Jochum et al.^S1^ | 1006 | 48 | 16 |
| Sr/Ca | 342 | 4 * | ** | 341 | 2 | -0.4 | 6640 | 170 | ** | 6759 | 121 | 1.8 | 6670 | 230 | Jochum et al.^S1^ | 7076 | 133 | 6 |
| Ba/Ca | 683 | 7 * | ** | 687 | 5 | 0.6 | 59.6 | 1.4 | ** | 58.7 | 1.3 | -1.5 | 6.97 | 0.59 | Jochum et al.^S1^ | 8.84 | 2.72 | 26.9 |

* uncertainty

** GeoReM database (version 27) preferred values

*** calculated from the preferred value for MgO given in the GeoReM database.

**Supplementary Information reference**

S1. Jochum, K. P. *et al.* Nano-Powdered Calcium Carbonate Reference Materials: Significant Progress for Microanalysis? *Geostand. Geoanal. Res.* **43**, 595–609 (2019).

**
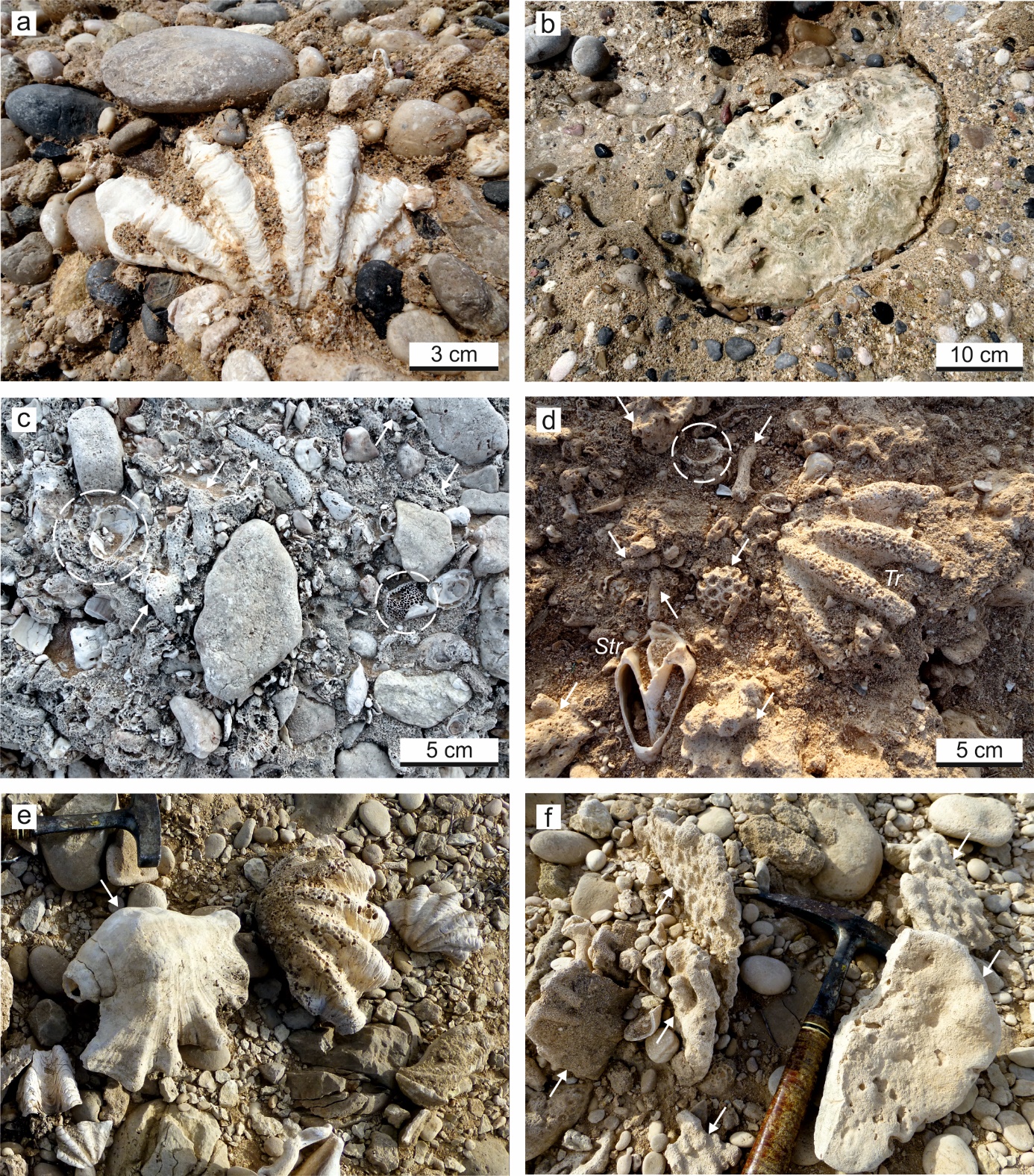
**

**Supplementary Figure S1.** *Tridacna*-bearing shallow-marine deposits in northeastern Oman. **(a, b)** Calcarenitic conglomerate containing disarticulated *Tridacna* shells (a) and rounded colonies of massive-growing *Porites* corals (b) at Dibab locality. **(c)** Coarse bioclastic conglomerate with abundant *Acropora* branch fragments (white arrows), balainid (dashed circles) and mollusk shell debris at Fins locality. **(d)** Coarse bioclastic, pebbly calcarenite at Sur locality. *Tridacna* (*Tr*) occurs associated with diverse reef corals (white arrows), balanids (dashed circles), and strombid gastropods (*Str*). **(f, e)** Weakly lithified conglomerate at Tiwi locality. Fossils found in the conglomerate include isolated *Tridacna* shells (e), large *Lambis* gastropods (e, white arrow) and rounded fragments of tabular *Acropora* corals (f, white arrows); hammer for scales.

**
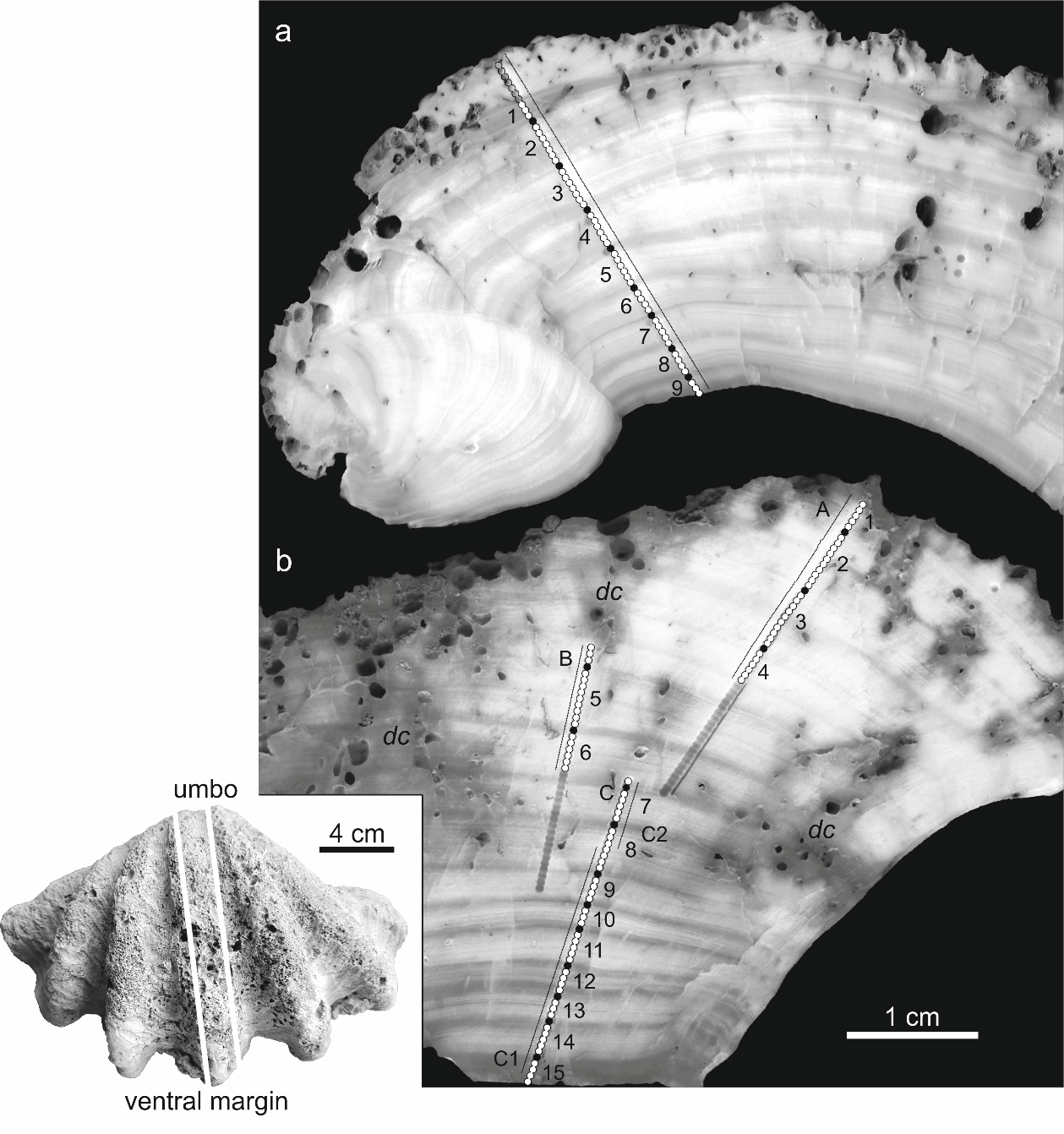
**

**Supplementary Figure S2.** Growth banding pattern in reflected light and sampling paths for stable isotope (beaded lines) and trace element (dotted thin lines) analyses. **(a)** T-Tiwi1. **(b)** T-Fins. Black dots in the stable isotope sampling paths mark δ^18^O maxima at the beginning of a δ^18^O cycle and open dots represent samples obtained from the outer shell layer, which are not considered in the statistics and SST estimates. Numbers refer to oxygen isotope cycles (years) in the stable isotope profiles (Figs. 3, 4). In T-Fins, only samples which are used for the composite record are indicated. A, B, C, C1 and C2 in (b) designate the individual sampling transects. Near borings and cracks, and at the outer surfaces the originally white shell of the studied specimens has locally turned diffuse grey due to the replacement of the primary aragonite by diagenetic calcite (*dc*), as revealed by microscopic inspection and X-ray diffraction analysis, and consequently the primary growth banding pattern is obscured within these areas. The slabs used for geochemical analysis (a, b) were cut from the shells along the axis of maximum growth (from the umbo to the ventral margin; left lower corner inset).


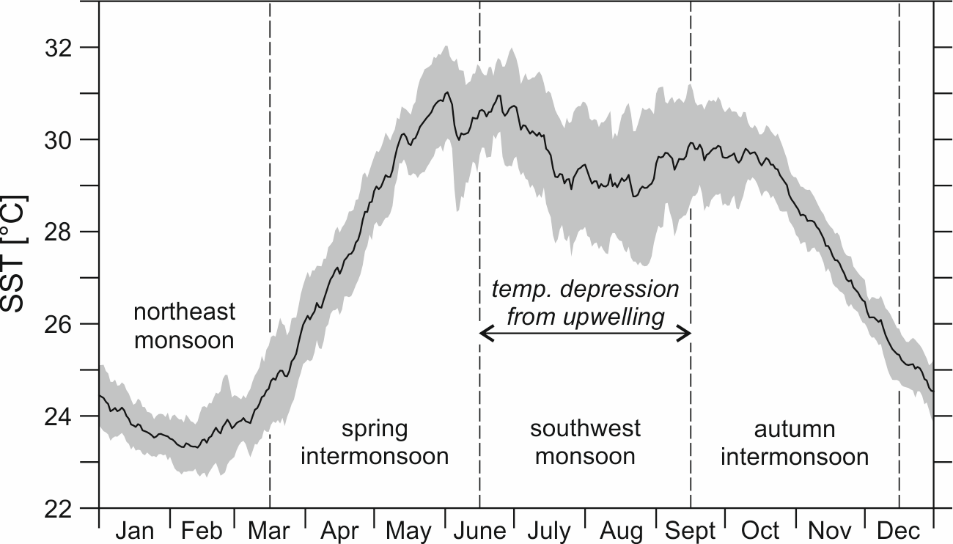


**Supplementary Figure S3.** Recent annual sea surface temperature cycle and monsoon seasons. Daily satellite SST data (JPL MUR MEaSUREs Project, https://doi.org/10.5067/GHGMR-4FJ04) for coastal waters off the village Tiwi in northeastern Oman (grid cell: N 22°50'24'', E 59°16'12'', spatial resolution 0.01°; Fig. 1c) shows an annual mean average of 27.6 ± 0.2 °C by a mean seasonality of 9.5 ± 0.8 °C in the years from 2003 to 2019. The warmest month is June, with a monthly average SST of 30.5 ± 0.7 °C, and the coldest is February with a monthly average of SST of 23.5 ± 0.5°C during the period under consideration. June has also the most daily sunshine hours, while the month with the fewest sunshine hours and most precipitation (18 mm) is January (https://de.climate-data.org/asien/oman/maskat/maskat-2089/). During the strongest SWM (July−August), the northeastern coast of Oman is not directly affected by coastal upwelling, but filaments of cool and productive water can spread into the Gulf of Oman from the Arabian Sea via moving gyres and eddy systems (Fig. 1b) accompanied by cloud cover^31,32^. Peak primary productivity in the Gulf of Oman occurs, however, during winter due to convective mixing of the water column^32^.


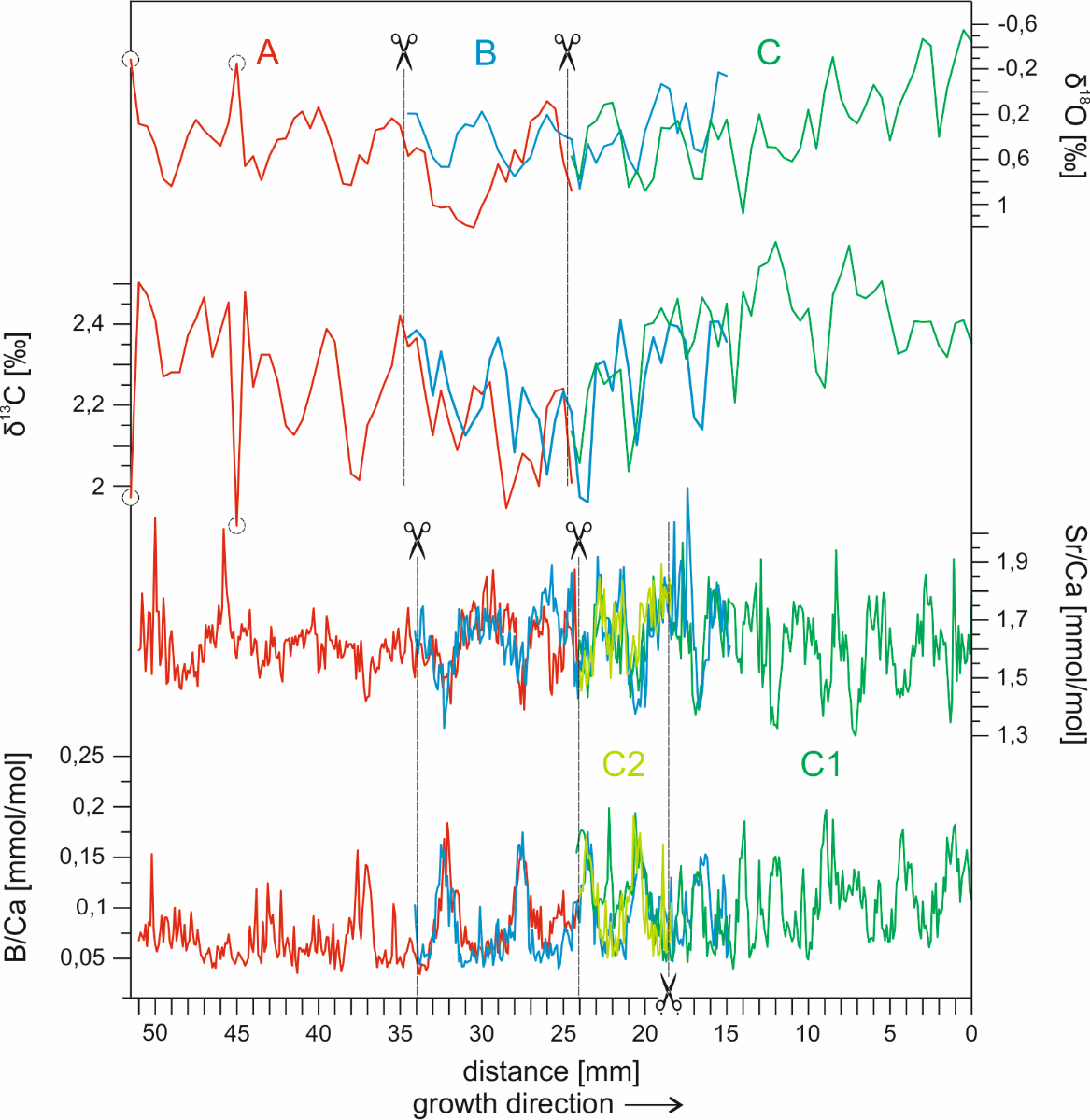


**Supplementary Figure S4.** T-Fins stable isotope and trace element composite record. The scissors symbol mark points at which the overlapping sub-records A, B, C, C1 and C2 (Supplementary Fig. S2) are cut and merged to a continuous record, and dashed circles indicate diagenetically altered stable isotope values, which are excluded from this composite record, statistics and SST estimation.
